# Global spatio-temporal evolution and health inequalities in high BMI-associated kidney cancer burden from 1990 to 2021 and burden prediction to 2040

**DOI:** 10.3389/fonc.2025.1630856

**Published:** 2025-12-18

**Authors:** Yawen Lu, Siwei Zhang, Jianfeng Ma, Yang Hu, Yiming Liu, Kun Zhang, Xinyu Luo, Xiaojuan He, Yirong Kong, Xue Han, Yongfeng Wang, Sheng Li, Haiyang Li

**Affiliations:** 1 The First Clinical Medical College of Lanzhou University, Lanzhou, Gansu, China; 2 Department of Digestive Disease, Weinan Central Hospital, Weinan, Shaanxi, China; 3 School of Stomatology Lanzhou University, Lanzhou, Gansu, China; 4 School of Pharmacy Lanzhou University, Lanzhou, Gansu, China; 5 The First Hospital of Lanzhou University, Lanzhou, Gansu, China; 6 Urology Department of Gansu Provincial People's Hospital, Lanzhou, Gansu, China

**Keywords:** kidney cancer, high body-mass index, global burden, joinpoint regression analysis, Age-Period-Cohort model, future prediction

## Abstract

**Background:**

The primary cancer of the urinary system, kidney cancer is becoming more common worldwide and is linked to a high body mass index (BMI). Although 20% of kidney cancer cases are caused by obesity, current research data on the global burden of the disease and its trends across population groups are scarce, especially as predicted by 2040.

**Method:**

We examined age-standardized mortality rates (ASMR), disability-adjusted life years (DALYs), and sociodemographic index (SDI) using Global Burden of Disease (GBD 2021) data from 204 nations and territories. joinpoint regression revealed changes in temporal trends and age-period-cohort (APC) modeling separated the effects of age, period, and cohort. Finally, we project the disease burden to 2040.

**Result:**

From 1990 to 2021, high BMI-related kidney cancer deaths increased by 2.67-fold, and DALYs rose by 66.1%. In 2021, the ASMR for high BMI-associated kidney cancer was 0.38 (95% per 10 0,000 UI: 0.12-0.52) and the ASDR was 8.99 per 100,000 (95% UI: 3.68-14.51). Significant heterogeneity was observed in gender and age, with a significantly higher male burden concentrated in the 55–79 year group. The main burden is concentrated in the high SDI region, including South Latin America, North America, Europe and North Asia. Over 30 years, the burden of high BMI-associated kidney cancer gradually increased, especially in low SDI areas, while high SDI areas showed a decreasing trend after 2016. The global disease burden of high BMI-associated kidney cancer burden grew fastest between 2000 and 2010, began to decline in 2016, and will rebound in 2030.

**Conclusion:**

The global burden of high BMI-associated kidney cancer burden has surged since 1990. Although it showed a downward trend in 2016, it is expected to rebound by 2030. Significant differences exist across regions, genders, and age groups. Policymakers must prioritize obesity prevention, adopt gender-specific strategies, enhance early detection in older populations, and address issues of socioeconomic inequality and unequal distribution of healthcare resources to tackle this public health challenge.

## 1 Introduction

As the 12th most frequent cancer globally, kidney cancer is a common urological malignancy that accounts for 2.2% of all cancer incidence and 1.8% of all cancer mortality (1). The World Health Organization (WHO) International Agency for Research on Cancer’s most recent statistics from 2020 shows that there are approximately 431,000 new instances of kidney cancer reported globally, and that number is rising annually. Its incidence and death exhibit intricate and varied worldwide epidemiological patterns, with notable regional, age-, and gender-specific variations. Kidney cancer’s disease burden is a serious worry.

Excess body fat is a hallmark of obesity, which is brought on by intricate interplay between behavioral, environmental, genetic, and socioeconomic factors (2). The body mass index (BMI) is a useful metric for determining total body fat. Adults with a BMI of 25-29.9 kg/m^2^ were considered overweight, while those with a BMI over 30 kg/m^2^ were considered obese. From 1990 to 2022, the global age-standardized prevalence of obesity rose markedly—from 8.8% to 18.5% in adult women, 4.8% to 14.0% in adult men (3). Mechanistically, obesity promotes cancer development through chronic inflammation, insulin resistance, dysregulated adipokine secretion, and gut microbiota dysbiosis (4), and a weight loss of more than 5 kg has been shown to reduce overall cancer risk (5). Studies (6–8) have shown that obesity is a major risk factor for renal cancer and have proposed the concept of the obesity paradox. Given the continuing rise in global obesity rates, understanding the impact of obesity on the burden of renal cancer is critical.

Although there are many research on the clinical management of kidney cancer, the disease’s worldwide disease burden has received relatively less attention, and there is a dearth of longitudinal analysis of changes in the burden of kidney cancer. Furthermore, despite the fact that prior research has assessed the influence of particular risk factors, such as occupational exposure to trichloroethylene (9), on the burden of kidney cancer, there is still a dearth of thorough, systematic studies on the burden resulting from high BMI globally, particularly lacking the analysis of disease burden and sociodemographic index (SDI), cohort study and future prediction. This implies that more accurate techniques are required to measure and examine variations in disease load and health outcomes across time periods and demographic groupings in future.

The purpose of this study is to investigate in detail the global burden of kidney cancer as determined by the Global Burden of Disease (GBD 2021) study, as well as trends in high BMI in various categories from 1990 to 2021, including age, gender, SDI quintiles, GBD subcontinent regions, and countries. Using an age-cycle cohort model, we also conducted a combined point regression to examine the disease’s temporal patterns over the previous 30 years. Finally, we project the disease burden to 2040. These results offer important information for the creation of policies and initiatives targeted at reducing obesity worldwide, preventing kidney cancer, and safeguarding high-risk populations.

## 2 Materials and methods

### 2.1 Data source

This study uses the GBD 2021 database to describe the global burden of illness based on kidney cancer with a high BMI. GBD 2021 uses data from national vital statistics, cancer registries, autopsy reports of death, national health surveys, census, and published research to evaluate disease mortality, disability, and 88 risk factors (10). It covers 23 age groups from 1990 to 2021, all genders, and 204 nations and regions. Kidney cancer cases were defined according to the International Classification of Diseases, Tenth Revision (ICD-10) code C64, encompassing malignant neoplasms of the kidney excluding renal pelvis (C65) and ureter (C66). Data were restricted to primary kidney cancers, excluding metastatic cases to ensure specificity in burden estimation. Using high BMI as a risk factor, we were able to get pertinent data on kidney cancer. We also acquired estimates of the number of fatalities and disability-adjusted life years (DALYs) from kidney cancer linked to high BMI, along with other epidemiological data. Each piece of data used in this study is available to the public and the dataset link is https://ghdx.healthdata.org/gbd-results-tool.

### 2.2 Basic study variables

Estimates of the burden of kidney cancer, including deaths and DALYs. Years Lived with Disability (YLDs) and Years of Life Lost (YLLs) were added up to determine DALYs. For the GBD 2021-year standard population, age-standardized rates (mean rate per 100,000 individuals) were computed using direct standardized procedures. All age-standardized mortality (ASMR) and age-standardized DALY rates (ASDR) values were age-corrected (11). Income, education, and fertility rates are combined to create SDI, which ranges from 0 to 1 and is also a measure of social and national progress (12). The degree of development increases with the value. These extensive data sets offer a strong starting point for researching the worldwide incidence of kidney cancer brought on by high BMI.

### 2.3 Attributable burden calculation

The GBD study uses a comparative risk assessment (CRA) approach to quantify how much disease burden can be linked to high BMI. The link between high BMI and kidney cancer draws on meta-analyses of epidemiological data that report relative risks (RRs), adjusted for key confounders such as smoking, alcohol consumption, genetic susceptibility, and hypertension. These adjusted RRs are then mapped onto population BMI distributions to derive the population attributable fraction (PAF). The PAF was used to estimate the burden of kidney cancer attributable to high BMI. The formula for PAF is:


$$PAF=\frac{\sum \left(P_{i}\times \left(RR_{i}-1\right)\right)}{1+\sum \left(P_{i}\times \left(RR_{i}-1\right)\right)}$$

where $P_{i}$ is the prevalence of high BMI in the i-th age group, and $RR_{i}$ is the relative risk of kidney cancer associated with high BMI in that age group. Attributable deaths and DALYs were calculated by multiplying total deaths and DALYs by PAF.

### 2.4 Study analysis

R software was used for all statistical analyses, and p< 0.05 was chosen as the threshold for statistical significance. The indicators will calculate 95% uncertainty interval (95% UI) or 95% confidence interval (95% CI) to ensure the accuracy of the estimation results.

#### 2.4.1 Preliminary analysis

In order to investigate temporal trends in kidney cancer burden, we employed a least-squares linear regression model and the estimated annual percentage change (EAPC) as a tool to assess changes in the Age-Standardized Rate (ASR) over a specific time period (13). EAPC is 100 (exp (β) -1), where β is the coefficient of the linear regression model ln (ASR) =α+βx+ϵ, and year is denoted by x. When the EAPC index and its 95% CI are larger than zero, the ASR trend is regarded as upward; when they are less than zero, the ASR trend is seen as downward. These patterns are regarded as steady otherwise.

We created a worldwide map and carried out a regional comparative study in order to examine the regional variations and global distribution of kidney cancer depending on high BMI. To display the regional distribution of illness burden, data from the geographic regions identified by the GBD study were combined, and maps were produced using the R software’s ggplot2 and sf tools (version 4.3.3). In order to investigate the prevalence of kidney cancer with high BMI in various demographic groups, such as age, sex, and certain subgroups, we also conducted population-based studies. Age groupings were used to stratify the data for both men and female. R was used for all statistical analyses, and the ggplot2 software package was used to show the findings. Additionally, we compared the disease burden at various socioeconomic development levels to examine the association between SDI and kidney cancer burden with high BMI. To further quantify socioeconomic-related health inequalities, we employed two established metrics: the Slope Index of Inequality (SII) and the Concentration Index (CIx). The SII is an absolute measure of inequality, calculated by performing a weighted least squares regression of health outcomes on the relative socioeconomic rank (based on SDI). The CIx is a relative measure derived from the concentration curve (a variant of the Lorenz curve), which plots the cumulative proportion of the health outcome against the cumulative proportion of the population, ranked by SDI from lowest to highest. R’s dplyr and ggplot2 software tools are mostly used for data processing and visualization.

#### 2.4.2 Joinpoint regression analysis

Joinpoint regression is a statistical technique used to analyze time series data. It identifies “joinpoints,” which represent significant shifts or inflection points in trends over time. This model offers a useful tool for evaluating the trajectory of these markers over time in research examining the impact of high BMI on deaths, DALYs, YLLs, and YLDs in kidney cancer. First, testing the null hypothesis H0: no connectivity point. This suggests that there was no significant trend change during the study period, and changes in kidney cancer related variables were initially considered to be linear. Then, setting 3 turning points greatly improved the fit of the model. In essence, the goal of this phase is to find time points that may indicate a significant change in the trend. A Monte Carlo permutation test (α=0.05) was subsequently performed, the annual percentage change (APC) and average annual percentage change (AAPC) was calculated for each fragment. To determine whether the difference between models with different numbers of connections is statistically significant if the null hypothesis is rejected, suggesting at least one meaningful connection. This procedure helps to determine which model best captures the data trends.

APC, AAPC, and its 95% CI were important outcome indicators (14). While the AAPC shows the average annual rate of change throughout the whole study period, the APC shows the average annual rate of change in kidney cancer burden for a specific time period. Based on whether the 95%CI of APC is completely above or below 0 or contains 0, the trend is up, down or not significant.

#### 2.4.3 Age-Period-Cohort model

An essential statistical technique for resolving trends (15) in time series data and differentiating the effects of cohort, age, and period on the outcome variables is the Age-Period-Cohort model. Building a multivariate regression model is the fundamental idea behind the Age-Period-Cohort model:


$$Y_{t}=\beta_{0}+\beta_{1}\;Age{\;}_{t}+\beta_{2}\;Period{\;}_{t}+\beta_{3}\;Cohort{\;}_{t}+\epsilon_{t}$$

${\text{Y}}_{\text{t}}$ represents the outcome variables on the ${\text{ Age }}_{\text{t}}$, ${\text{ Period }}_{\text{t}}$ or ${\text{Cohort }}_{\text{t}}$. ${\text{ Age }}_{\text{t}}$, ${\text{ Period }}_{\text{t}}$ or ${\text{Cohort }}_{\text{t}}$ represents the outcome variables on the $\;\;\beta_{1}$, $\beta_{2}$ and $\beta_{3}$. These lines quantified the independent effect of each factor on the outcome variable. $\beta_{0}$ is an intercept term representing the expected value of the outcome variable where all independent variables are zero. $\epsilon_{t}$ is the error term, reflecting the variation that is not explained by the model. The Age-Period-Cohort model assumes linear cohort effects, which may not fully capture the complex, non-linear interactions between obesity and renal carcinogenesis.

The age group and observation period were split into conventional 20-year and 5-year intervals (16). The number of deaths and DALYs were independently examined, with age effects modeled as 5-year intervals (20–24 to 95 + years), period effects as 5-year span (1990–1994 to 2015-2021), and birth cohorts from the age-period combination. To reveal the dynamic characteristics of the relationship between high BMI and kidney cancer, we will specifically assess the changing trends in kidney cancer burden across different age groups, time periods, and birth cohort. Thus, it not only provides insight into the effects of high BMI on the age, period and cohort effects of renal cancer, but also for the development of targeted prevention and intervention strategies. The intrinsic estimation method was used to fit the Age-Period-Cohort model to solve the collinearity problem. The sensitivity analysis excluded outliers (e. g., areas with<50 cases per year) to ensure robustness.

#### 2.4.4 Frontier analysis

Frontier analysis is a quantitative assessment method that models the nonlinear relationship between the SDI and disease burden to determine the lowest achievable disease burden at a given level of social development, thereby evaluating the potential for improvement in health performance across different regions. In this study, we examined the link between kidney cancer burden and high BMI, measuring ASMR and ASDR relative to SDI-based development levels. The frontier represents the lowest achievable rates for a given SDI, with deviations indicating unrealized reductions in fatalities and disability. Using 1990–2021 data, we constructed a nonlinear frontier via the free disposal hull method, excluding outliers. Bootstrapping (100 samples) addressed uncertainty, while LOESS regression (degree 1, span 0.2) smoothed the frontier. Effective difference—the gap between a region’s 2021 rates and the frontier—quantified potential improvements. Regions outperforming the frontier received a zero-distance score.

#### 2.4.5 Future prediction

The Bayesian age-period-cohort (BAPC) model, developed by Riebler and Held, reliably predicts the future burden of high BMI-associated kidney cancer. This advanced statistical tool incorporates age, period, and cohort effects, offering stable estimates even with limited data. Using integrated nested Laplace approximations, we integrated GBD 2021 data to forecast death and DALY rates, with predictions based on obesity trends. The model avoids Markov chain Monte Carlo issues, outperforming linear models by providing broader age, sex, and regional coverage and accurate 95% CI.

In our study, we harnessed a rich trove of historical information that reached back to 1990 and stretched all the way to 2021. We concentrated our efforts on understanding the global reach of kidney cancer, examining sociodemographic patterns, and noting the escalation in cases associated with high BMI. This comprehensive approach allowed us to factor in the effects of an aging population and disparities in obesity management across regions, all while predicting future trends in the current epidemiological climate. By harmonizing our methods with well-established protocols, we aimed to deliver a reliable and cohesive estimation of the global toll of kidney cancer associated with high BMI levels.

## 3 Results

### 3.1 Global distribution and trends in high BMI-associated kidney cancer from 1990 to 2021

As the global prevalence of high BMI escalates, kidney cancer resulted in 32,362.97 (95% UI: 13,158.99–52,690.56) deaths worldwide in 2021, representing a 2.67-fold increase compared to 1990 (12,111.53, 95% UI: 4,755.11–19,779.84) (**Table 1**). The total DALYs attributed to high BMI-related kidney cancer reached 781,627.21 (95% UI: 319,389.34–1,260,313.35) in 2021 (**Table 2**). The global ASMR for high BMI-associated kidney cancer rose from 0.32 (95% UI: 0.12–0.52) per 100,000 in 1990 to 0.38 (95% UI: 0.15–0.62) in 2021, reflecting an EAPC of 20.07% (95% CI: 13.49–26.71) over three decades. Similarly, the ASDR increased from 7.8 (95% UI: 3.06–12.69) to 8.99 (95% UI: 3.68–14.51), with an EAPC of 15.35% (95% CI: 8.83–22.08) (**Tables 1** and **2**).

**Table 1:** Deaths cases and ASMR per 100,000 population of high BMI-associated kidney cancer in 1990 and 2021.

Characteristics	1990		2021		1990-2021
	Deaths.Cases.No.(95% UI)	ASMR per 100,000 No.(95% UI)	Deaths.Cases.No.(95% UI)	ASMR per 100,000 No.(95% UI)	EAPC(%) in ASMR No.(95% CI)
Global	12111.53 (4755.11,19779.84)	0.32 (0.12,0.52)	32362.97 (13158.99,52690.56)	0.38 (0.15,0.62)	20.07 (13.49,26.71)
Central Europe, Eastern Europe, and Central Asia	3105.14 (1209.92,5023.35)	0.64 (0.25,1.04)	6389.47 (2616.38,10279.83)	0.97 (0.4,1.56)	50.62 (35.47,63.55)
High-income	7212.85 (2834.12,11745.96)	0.6 (0.24,0.98)	15290.38 (6174.42,24498.99)	0.68 (0.28,1.09)	13.04 (7.21,19.78)
Latin America and Caribbean	605.89 (238.65,994.08)	0.27 (0.11,0.44)	3223.57 (1321.34,5276.25)	0.52 (0.21,0.85)	91.05 (75.77,109.45)
North Africa and Middle East	276.53 (109.31,443.99)	0.17 (0.07,0.27)	1458.91 (618.34,2323.32)	0.33 (0.14,0.53)	97.59 (67.76,135.13)
South Asia	113.47 (42.33,182.2)	0.02 (0.01,0.03)	903.33 (333.79,1475.84)	0.06 (0.02,0.1)	207.38 (152.92,267.43)
Southeast Asia, East Asia, and Oceania	668.76 (260.15,1107.03)	0.06 (0.02,0.1)	4457.68 (1656.06,7651.75)	0.16 (0.06,0.27)	160.37 (110.01,216.09)
Sub-Saharan Africa	128.9 (47.12,206.21)	0.06 (0.02,0.1)	639.63 (235.57,1029.69)	0.14 (0.05,0.22)	120.01 (96.82,144.37)
Gender					
Male	7091.37 (2752.82,11606.69)	0.41(0.16, 0.67)	20508.83(8301.87,33298.13)	0.53(0.21, 0.86)	0.29(0.20, 0.38)
Female	5020.16 (1973.79,8218.53)	0.24(0.09, 0.39)	11854.14(4785.72,19033.20)	0.26(0.10, 0.41)	0.06(-0.00, 0.13)
Socio-demographic Index (SDI)					
Low SDI	84.16 (31.56,136.26)	0.04 (0.01,0.06)	380.98 (133.8,636.17)	0.07 (0.03,0.12)	101.37 (68.4,143.81)
Low-middle SDI	281.88 (108.98,456.35)	0.05 (0.02,0.08)	1671.49 (661.24,2719.27)	0.12 (0.05,0.19)	149.8 (123.65,179.08)
Middle SDI	984.01 (379.89,1603.94)	0.1 (0.04,0.16)	5552.09 (2283.85,9134.25)	0.21 (0.09,0.34)	114.08 (93.47,134.87)
High-middle SDI	3970.99 (1559.38,6490.61)	0.4 (0.16,0.65)	10262.9 (4130.5,16700.36)	0.52 (0.21,0.84)	29.06 (17.79,39.17)
High SDI	6766.78 (2660.27,11032.72)	0.61 (0.24,1)	14445.48 (5897.31,23280.46)	0.67 (0.28,1.08)	9.75 (3.76,15.97)

**Table 2:** DALYs cases and ASDR per 100,000 population of high BMI-associated kidney cancer in 1990 and 2021. .

Characteristics	1990		2021		1990-2021
	DALYs.Cases.No.(95% UI)	ASDR per 100,000 No.(95% UI)	DALYs.Cases.No.(95% UI)	ASDR per 100,000 No.(95% UI)	EAPC(%) in ASDR No.(95% CI)
Global	318069.51(125099.3,517059.58)	7.8 (3.06,12.69)	781627.21(319389.34,1260313.35)	8.99 (3.68,14.51)	15.35 (8.83,22.08)
Central Europe, Eastern Europe, and Central Asia	85540.87(33365.17,138158.91)	17.55 (6.83,28.33)	157099.93(64657.68,250619.18)	24.5 (10.08,39.03)	39.62 (25.11,52.42)
High-income	178706.84(70630.07,290033.34)	15.48 (6.12,25.11)	327728.21(134629.38,522645.43)	16.55 (6.83,26.25)	6.92 (1.05,13.18)
Latin America and Caribbean	17938.78(7089.65,29276.32)	7.38 (2.92,12.06)	88181.83(36433.59,143785.29)	13.83 (5.71,22.57)	87.42 (71.67,106.12)
North Africa and Middle East	8188.49(3244.81,13231.77)	4.39 (1.74,7.08)	41675.37(17783.52,65829.71)	8.37 (3.56,13.33)	90.68 (63.23,123.99)
South Asia	3396.35(1265.28,5504.12)	0.53 (0.2,0.85)	25771.84(9506.27,41702.1)	1.62 (0.6,2.63)	207.9 (155.17,263.97)
Southeast Asia, East Asia, and Oceania	20485.8(8008.8,33722.47)	1.63 (0.64,2.69)	122344.39(44994.09,212060.73)	4.22 (1.56,7.32)	158.65 (104.81,217.94)
Sub-Saharan Africa	3812.39(1408.52,6076.05)	1.62 (0.6,2.6)	18825.64(7037.08,30668.44)	3.48 (1.29,5.6)	114.76 (89.8,143.44)
Gender					
Male	194671.12(75710.64,316754.04)	10.10(3.92, 16.46)	515011.67(209586.78,838321.79)	12.52(5.09, 20.38)	0.24(0.15, 0.32)
Female	123398.39(48939.63,200531.22)	5.74(2.27, 9.33)	266615.54(107491.92,422219.83)	5.81(2.34, 9.20)	0.01(-0.04, 0.08)
Socio-demographic Index (SDI)					
Low SDI	2506.25(939.16,4092.35)	0.99 (0.37,1.62)	11481.83(4012.38,19353.07)	1.99 (0.7,3.33)	100.42 (65.62,146.45)
Low-middle SDI	8458.45(3283.24,13698.54)	1.24 (0.48,2.02)	48209.57(19117.33,78003.09)	3.1 (1.23,5.03)	149.34 (122.45,178.48)
Middle SDI	29659.43(11466.4,48316.13)	2.57 (0.99,4.2)	154799.35(63281.93,253630.94)	5.5 (2.25,9.03)	113.87 (94.11,133.85)
High-middle SDI	108583.32(42625.81,177409.56)	10.51 (4.12,17.16)	253442.81(102934.78,411348.66)	12.88 (5.23,20.9)	22.53 (11.66,33.08)
High SDI	168242.98(66399.86,273509.14)	15.65 (6.18,25.42)	312528.87(129704.12,498459.29)	16.15 (6.7,25.64)	3.2 (-2.47,9.32)

Substantial disparities were observed across GBD regions. Central Europe, Eastern Europe, and Central Asia exhibited the highest ASMR in 2021 (0.97, 95% UI: 0.40–1.56), approximately 6.93 times greater than Sub-Saharan Africa (0.14, 95% UI: 0.05–0.22). The second is Latin America and Caribbean. Similarly, ASDR in Central Europe (24.50, 95% UI: 10.08–39.03) surpassed rates in South Asia (1.62, 95% UI: 0.60–2.63) and Southeast Asia, East Asia, and Oceania (4.22, 95% UI: 1.56–7.32). Gender-specific trends revealed higher absolute mortality and DALYs among males (20,508.83 deaths; 515,011.67 DALYs) compared to females (11,854.14 deaths; 266,615.54 DALYs) in 2021. Males also exhibited faster growth in age-standardized rates, with EAPCs of 0.29% (95% CI: 0.20–0.38) for ASMR and 0.24% (95% CI: 0.15–0.32) for ASDR, compared to females (ASMR EAPC: 0.06%, 95% CI: -0.00–0.13; ASDR EAPC: 0.01%, 95% CI: -0.04–0.08). Socio-demographic disparities were pronounced. The ASMR (0.67, 95% CI: 0.28,1.08) and ASDR (16.15, 95% CI: 6.7,25.64) are the largest in high SDI countries. High-income nations saw a 9.75% (95% CI: 3.76,15.97) rise in ASMR, while Low-middle SDI regions reported a 149.8% (95% CI: 123.65,179.08) increase. Notably, Latin America and the Caribbean exhibited the steepest ASMR growth (EAPC: 91.05%, 95% CI: 75.77–109.45), underscoring the escalating burden in transitioning economies (**Tables 1** and **2**).

From 1990 to 2021, the global age-standardized deaths rate for high BMI-associated kidney cancer increased from 0.23 (1990) to 0.41 per 100,000 (2021), marking a 78.3% rise (**Table 3**). Similarly, the DALYs rate escalated from 5.96 to 9.90 per 100,000, reflecting a 66.1% increase over three decades. Notably, YLLs dominated the DALYs burden, accounting for 95.7% of total DALYs in 2021 (YLLs rate: 9.47 vs. YLDs rate: 0.43), underscoring premature mortality as the primary driver of health loss (**Table 3**). Males consistently bore a higher burden across all metrics. In 2021, the male deaths rate (0.52) was 1.72 times higher than females (0.30), while the male DALYs rate (13.01) exceeded females (6.78) by 1.92-fold (**Table 3**). And, males exhibited faster relative growth in age-standardized rates. Male Deaths rates rose by 57.89% (0.19 to 0.30), compared to 100% in females (0.26 to 0.52). Male DALY rates increased by 79.30% (7.25 to 13.01), slightly outpacing males (45.50%, 4.66 to 6.78) (**Table 3**). While YLDs contributed minimally to total DALYs, the rate grew by 119.2% (0.20 to 0.43), indicating a rising prevalence of long-term disability from kidney cancer. YLLs mirrored the overall DALYs trend, increasing by 64.3% (5.77 to 9.47), driven by population aging and persistent gaps in early detection and treatment (**Table 3**).

**Table 3:** Temporal trends in deaths rate, DALYs rate, YLDs rate, and YLLs rate per 100,000 population for high BMI-associated kidney cancer from 1990 to 2021.

Year	Deaths rate(per 100,000)			DALYs rate(per 100,000)			YLDs rate(per 100,000)			YLLs rate(per 100,000)		
	Both	Male	Female	Both	Male	Female	Both	Male	Female	Both	Male	Female
1990	0.23	0.26	0.19	5.96	7.25	4.66	0.20	0.22	0.17	5.77	7.02	4.49
1991	0.23	0.27	0.19	6.09	7.42	4.75	0.20	0.23	0.17	5.89	7.18	4.58
1992	0.24	0.28	0.20	6.25	7.65	4.83	0.21	0.24	0.18	6.04	7.41	4.65
1993	0.25	0.29	0.20	6.46	7.95	4.95	0.22	0.26	0.19	6.24	7.69	4.77
1994	0.25	0.30	0.21	6.65	8.17	5.10	0.23	0.27	0.19	6.42	7.91	4.91
1995	0.26	0.30	0.21	6.73	8.29	5.15	0.24	0.28	0.20	6.49	8.01	4.95
1996	0.26	0.31	0.21	6.76	8.37	5.12	0.24	0.29	0.20	6.51	8.08	4.92
1997	0.26	0.31	0.21	6.82	8.46	5.15	0.25	0.29	0.20	6.57	8.17	4.94
1998	0.27	0.32	0.22	6.93	8.63	5.20	0.26	0.30	0.21	6.67	8.32	4.99
1999	0.27	0.33	0.22	7.04	8.81	5.24	0.26	0.31	0.21	6.77	8.49	5.03
2000	0.28	0.34	0.22	7.21	9.06	5.34	0.27	0.33	0.22	6.94	8.73	5.12
2001	0.29	0.34	0.23	7.36	9.26	5.44	0.28	0.34	0.23	7.08	8.92	5.21
2002	0.29	0.35	0.23	7.51	9.49	5.50	0.29	0.35	0.23	7.21	9.14	5.27
2003	0.30	0.36	0.24	7.68	9.75	5.59	0.30	0.36	0.24	7.38	9.38	5.35
2004	0.31	0.37	0.24	7.80	9.92	5.65	0.31	0.37	0.24	7.49	9.55	5.40
2005	0.31	0.38	0.24	8.00	10.22	5.76	0.32	0.39	0.25	7.68	9.83	5.51
2006	0.32	0.39	0.25	8.04	10.30	5.76	0.33	0.40	0.26	7.71	9.90	5.50
2007	0.32	0.39	0.25	8.12	10.44	5.78	0.34	0.41	0.26	7.79	10.03	5.51
2008	0.33	0.41	0.25	8.32	10.74	5.87	0.35	0.43	0.27	7.97	10.31	5.60
2009	0.34	0.41	0.26	8.45	10.91	5.96	0.36	0.44	0.28	8.09	10.47	5.68
2010	0.34	0.42	0.26	8.62	11.17	6.05	0.37	0.45	0.28	8.25	10.71	5.77
2011	0.35	0.43	0.26	8.72	11.32	6.09	0.37	0.46	0.29	8.34	10.86	5.81
2012	0.35	0.44	0.27	8.83	11.52	6.12	0.38	0.47	0.29	8.45	11.05	5.83
2013	0.36	0.45	0.27	8.97	11.71	6.20	0.39	0.48	0.29	8.58	11.23	5.90
2014	0.37	0.46	0.27	9.08	11.91	6.22	0.39	0.49	0.29	8.68	11.42	5.93
2015	0.38	0.47	0.28	9.24	12.14	6.31	0.40	0.50	0.30	8.84	11.64	6.01
2016	0.38	0.48	0.28	9.37	12.31	6.41	0.41	0.51	0.30	8.96	11.80	6.10
2017	0.39	0.49	0.28	9.41	12.36	6.43	0.41	0.51	0.31	9.00	11.85	6.13
2018	0.39	0.49	0.29	9.53	12.52	6.52	0.41	0.52	0.31	9.12	12.00	6.21
2019	0.40	0.50	0.29	9.67	12.73	6.59	0.42	0.52	0.31	9.25	12.20	6.28
2020	0.40	0.51	0.29	9.73	12.82	6.62	0.42	0.53	0.31	9.31	12.29	6.30
2021	0.41	0.52	0.30	9.90	13.01	6.78	0.43	0.54	0.32	9.47	12.47	6.46

### 3.2 Age and sex differences and temporal trends in kidney cancer burden associated with high BMI

**Figure 1:**
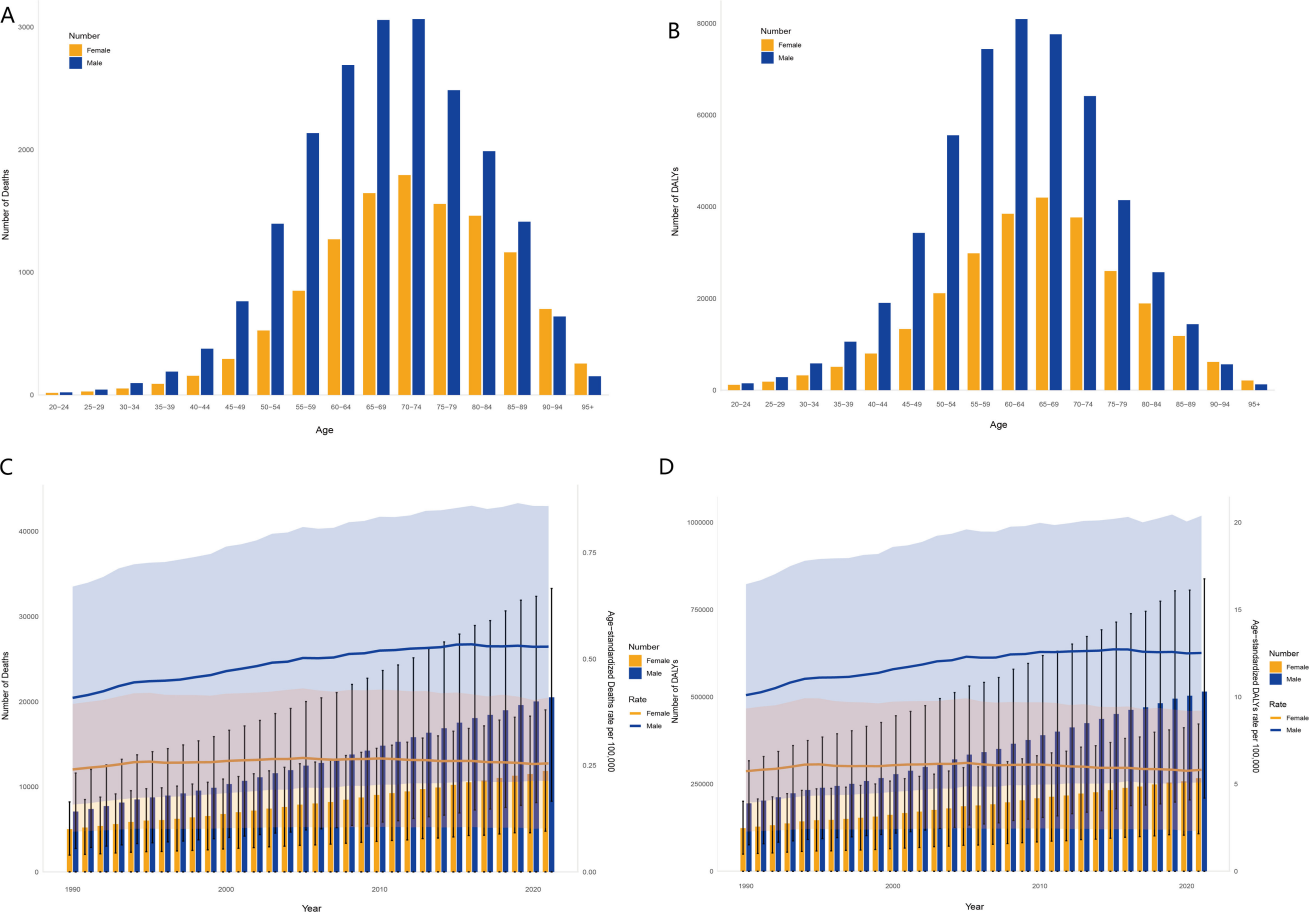
Age-sex characteristics and temporal patterns of the global burden of high BMI-associated kidney cancer. Number of deaths **(A)** and DALYs **(B)** across all age groups, 2021. Temporal trends of the number of deaths and ASMR **(C)**, and the number of DALYs and ASDR **(D)**, 1990–2021.

The global burden of high BMI-associated kidney cancer exhibited distinct demographic and temporal patterns. Death and DALY were consistently higher in male than in female in almost all age groups, and this difference was more pronounced in the 55–79 age group (**Figures 1A, B**). In 2021, the peak number of deaths associated with high BMI occurred in individuals aged 70–74 years, reflecting the heightened vulnerability of this age group (**Figure 1A**). Similarly, DALYs attributable to kidney cancer reached their highest levels in the 60–64 years age bracket for both sexes, demonstrating comparable trends between genders (**Figure 1B**). Overall, approximately 60% of deaths and DALYs were concentrated in individuals aged 60–79 years. The time analysis increased both deaths and the number of DALYs from 1990 to 2021. Annual kidney cancer-related deaths surged gradually, with a slight decline in 2016 (**Figure 1C**). DALYs also exhibited a consistent upward trajectory (**Figure 1D**).

**Figure 2:**
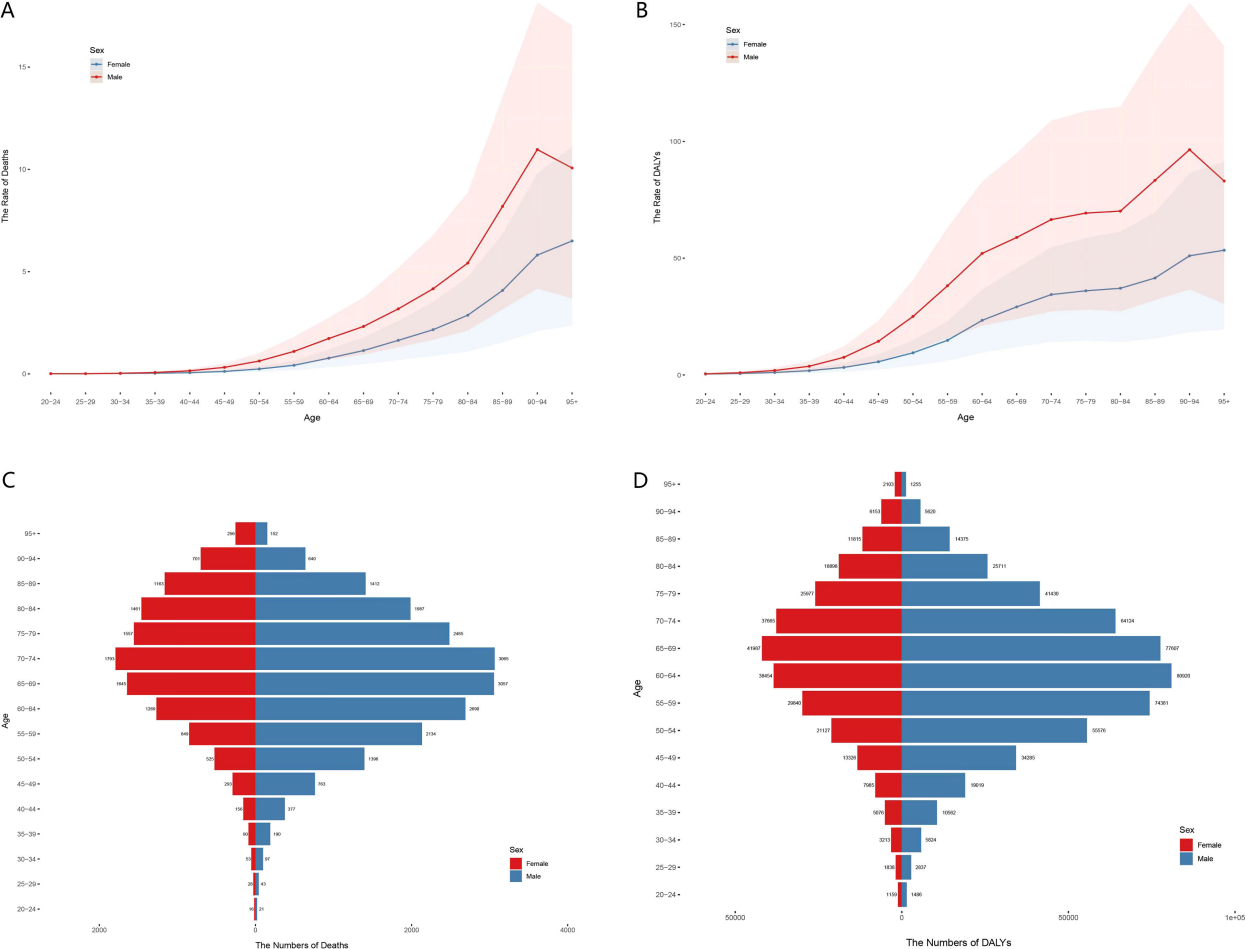
Age and sex differences in high BMI-associated kidney cancer in 2021. Deaths rates **(A)** and DALYs rates **(B)** for female and male in all age groups. The number of deaths **(C)** and DALYs **(D)** for female and male in all age groups.

Mortality and DALYs rates showed similar age and gender trends. From 20–24 years, female mortality and DALYs rates, gradually increased, and peaked in the 95+ -year age group. Male rose sharply, decreasing slightly after a peak in the 90–94 age group. Notably, female mortality and DALYs exceeded 2:1 in most age groups, highlighting the apparent gender imbalance (**Figures 2A, B**). Deaths increased with age, peaked in the 70–74 age group. In 2021, the age group of 70–74 years was 3065, compared with 1793 female in the same age group. Secondary peaks (3057 deaths were observed in male aged 60–64 years). Notably, deaths after the age of 85 (**Figure 2C**). DALYs from kidney cancer associated with high BMI were highest in the 60–64 age group (80920 DALYs) and the 65–69 age group (41987 DALYs) (**Figure 2D**).

### 3.3 Global burden analysis

**Figure 3:**
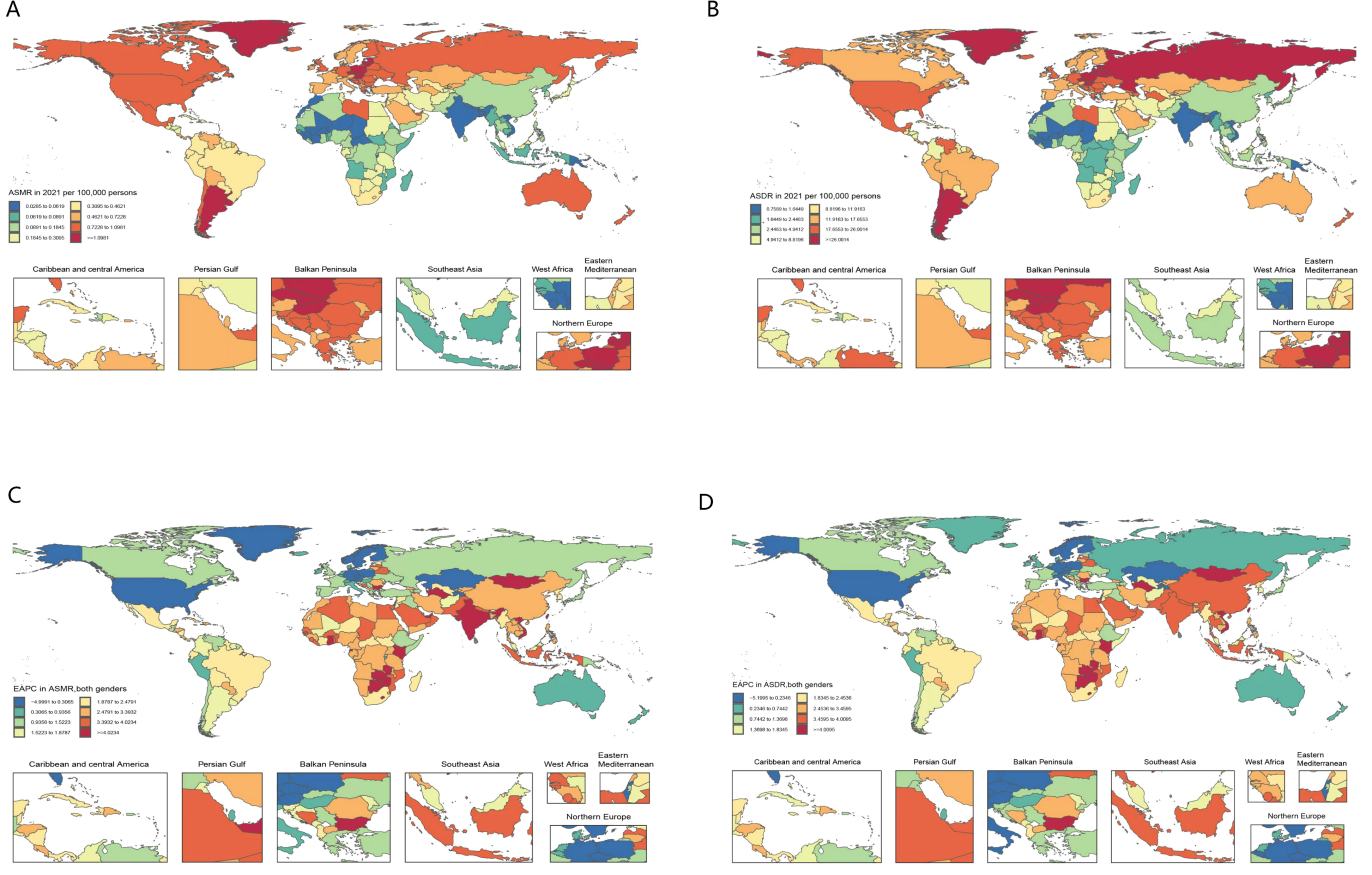
Global distribution of ASR of kidney cancer deaths and DALYs attributable to high BMI. ASMR **(A)** and ASDR **(B)** in 2021. The EAPC in ASMR **(C)** and ASDR **(D)**, 1990–2021.

There is significant geographical variation in the spatial distribution of ASMR and ASDR linked to high BMI in 2021. Countries such as South Latin America, North America, Europe and North Asia showed significantly higher ASMR and ASDR values, suggesting a greater kidney cancer burden in these regions associated with high BMI. In contrast, ASMR and ASDR values were lower, South, East and Southeast Asia, suggesting a relatively low impact of high BMI on kidney cancer in these regions (**Figures 3A, B**). The EAPC in ASMR and ASDR from 1990 to 2021 further highlights regional trends. In Africa, West Asia, South Asia, East Asia and Southeast Asia, indicating a significant trend in the kidney cancer burden from high BMI over the past 30 years. Conversely, EAPC was significantly decreased in North America, Europe, and North Asia, reflecting the decrease in kidney cancer burden associated with high BMI in these regions (**Figures 3C, D**).

### 3.4 Analysis of regional differences and health inequalities

**Figure 4:**
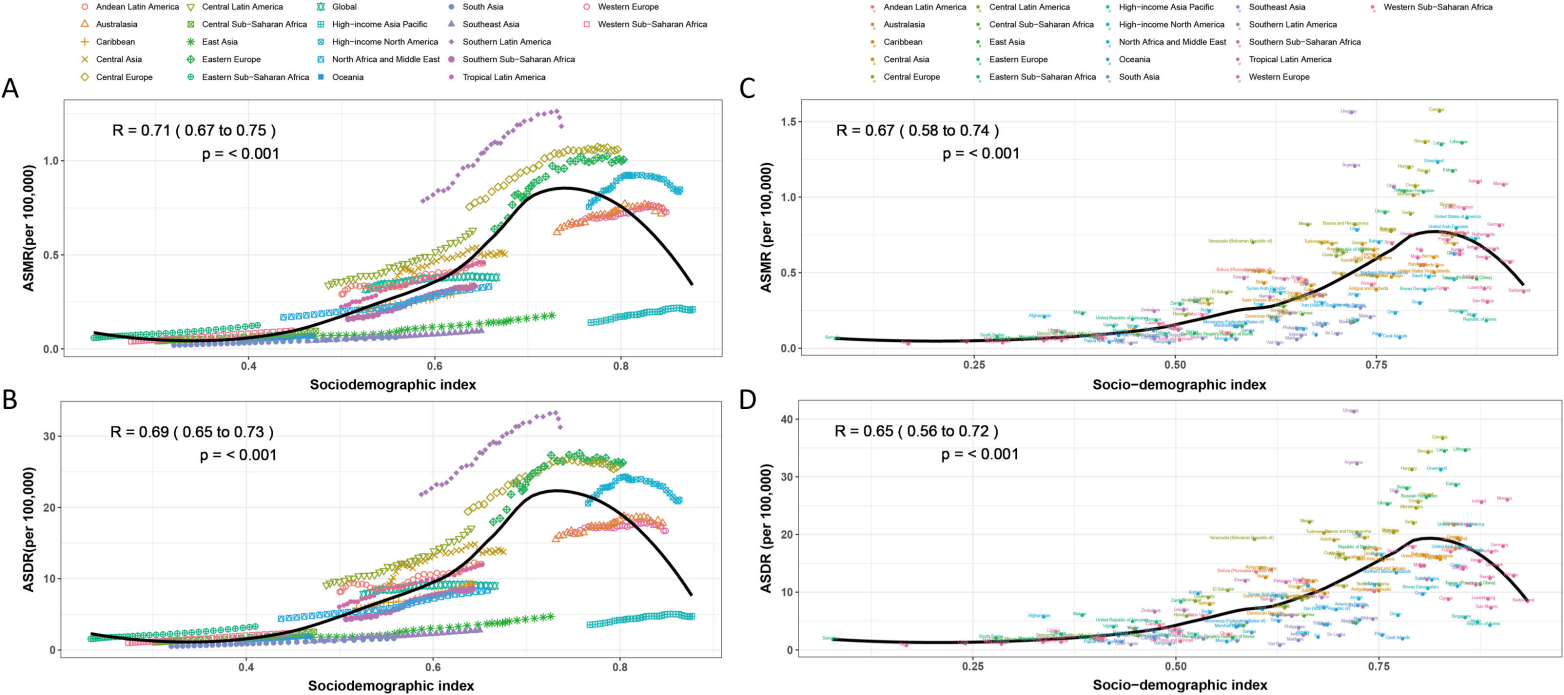
Correlation between ASMR, ASDR and SDI in high BMI-associated kidney cancer for 204 countries and 21 regions, 1990–2021. The above points show estimates for each country and region. The correlation between ASMR **(A)** or ASDR **(B)** and SDI in 21 GBD regions. The association between ASMR **(C)** or ASDR **(D)** and SDI in 204 countries.

The association between SDI and ASMR and ASDR of kidney cancer is non-linear both worldwide and within the 21 GBD regions. When SDI was< 0.75, the burden rose dramatically with increasing SDI. However, once they reached a particular SDI, they all began to decline. At the top of the curve (0.6–1.0 per 100,000), high-income regions like North America, Western Europe, and Oceania were concentrated. Conversely, the disease burden was lower in low SD locations (such as eastern and central sub-Saharan Africa). Keep in mind that the ASMR and ASDR values are significantly lower than those in other high SDI regions, even though the Asia Pacific region has high high-income SDI levels as well (**Figures 4A, B**).

At the national level, the association remained significant (R = 0.67, 95% CI: 0.58–0.74, p< 0.001). Countries with higher SDI (e.g., Japan, Germany) clustered toward higher ASMR values (0.5–1.0 per 100,000), contrasting with low-SDI nations in sub-Saharan Africa and South Asia (0.0–0.5 per 100,000). Heterogeneity was observed in Eastern Europe and Central Asia, where ASMR varied despite comparable SDI (**Figure 4C**). The correlation for ASDR was slightly weaker but significant (R = 0.65, 95% CI: 0.56–0.72, p< 0.001). High-SDI countries such as the United States and Australia exhibited ASDR values of 0.5–0.75 per 100,000, while low-SDI countries (e.g., Niger, Chad) consistently showed minimal burdens (<0.25 per 100,000) (**Figure 4D**).

**Figure 5:**
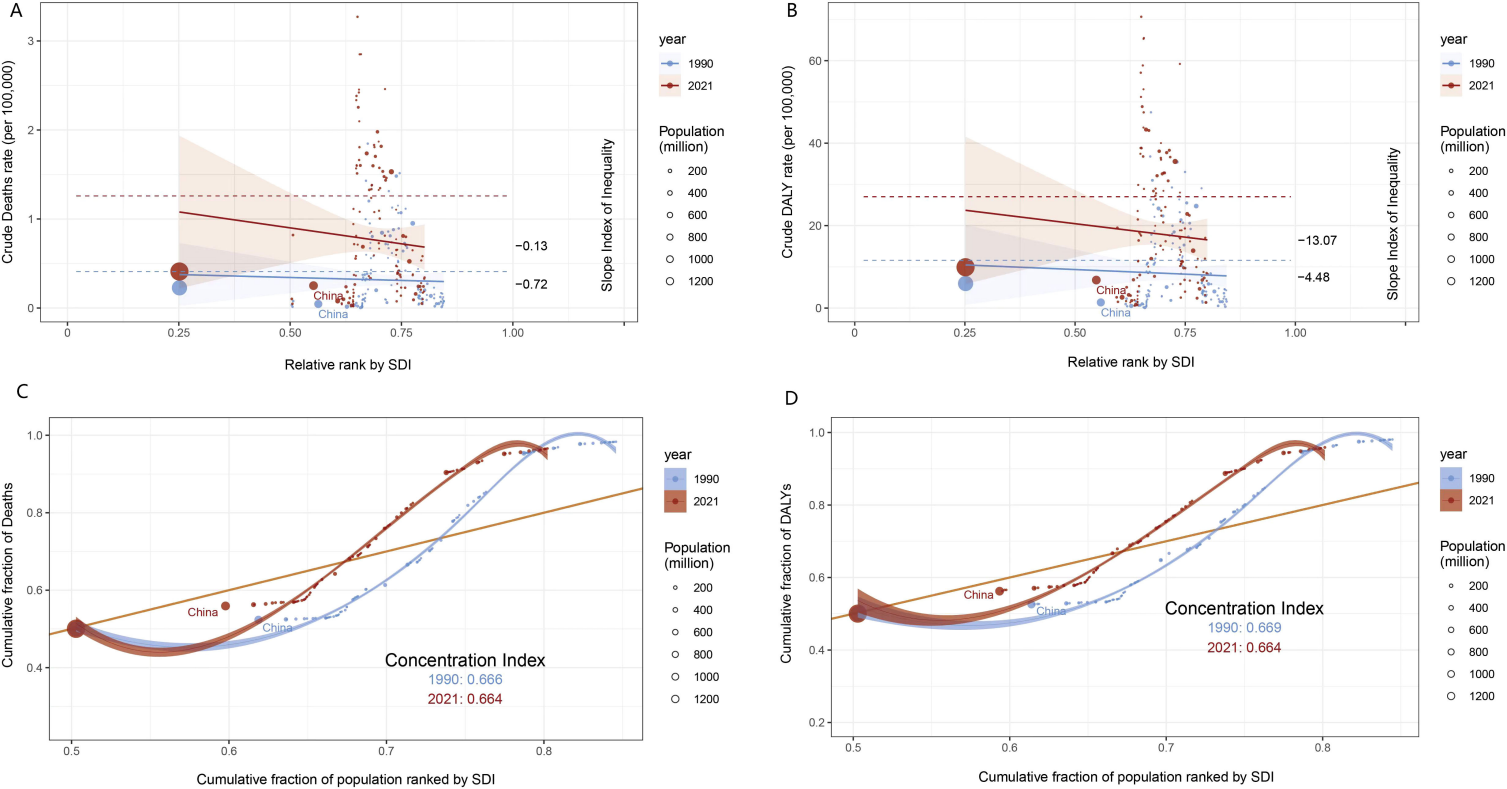
Analysis of deaths and DALYs inequality in kidney cancer associated with high BMI in 1990 and 2021. Crude deaths **(A)** and DALYs **(B)** rate per 100,000 by SDI, 1990 and 2021, by relative rank. Cumulative fraction of deaths **(C)** and DALYs **(D)** by cumulative population percentage, ordered by SDI in 1990 and 2021.

Globally, the burden of high BMI-associated kidney cancer was concentrated in low SDI areas between 1990 and 2021, and was consistently unequal among people with different SDI rankings. The health inequality index remained negative from 1990 to 2021 and decreased with the rise of SDI, indicating a greater burden of kidney cancer in areas with low SDI (**Figures 5A, B**). The SII for mortality rose from-0.72 in 1990 to-0.13 in 2021, indicating an improved inequality status (**Figure 5A**). The SII for the DALY rate decreased from-4.48 in 1990 to-13.07 in 2021, indicating a worsening inequality status (**Figure 5B**). The improvement in mortality is a positive signal, but the CIx of DALY warns that we cannot ignore the long-term impact of disease on people’s quality of life. The CIx of deaths and DALYs is 0.666 to 0.664 and 0.669 to 0.664 (**Figures 5C, D**), with less improvement. China has a large population base, with a significant increase in the distribution of mortality and DALYs from 1990 to 2021, making addressing health inequalities even more challenging and important.

### 3.5 Joinpoint regression analysis

**Figure 6:**
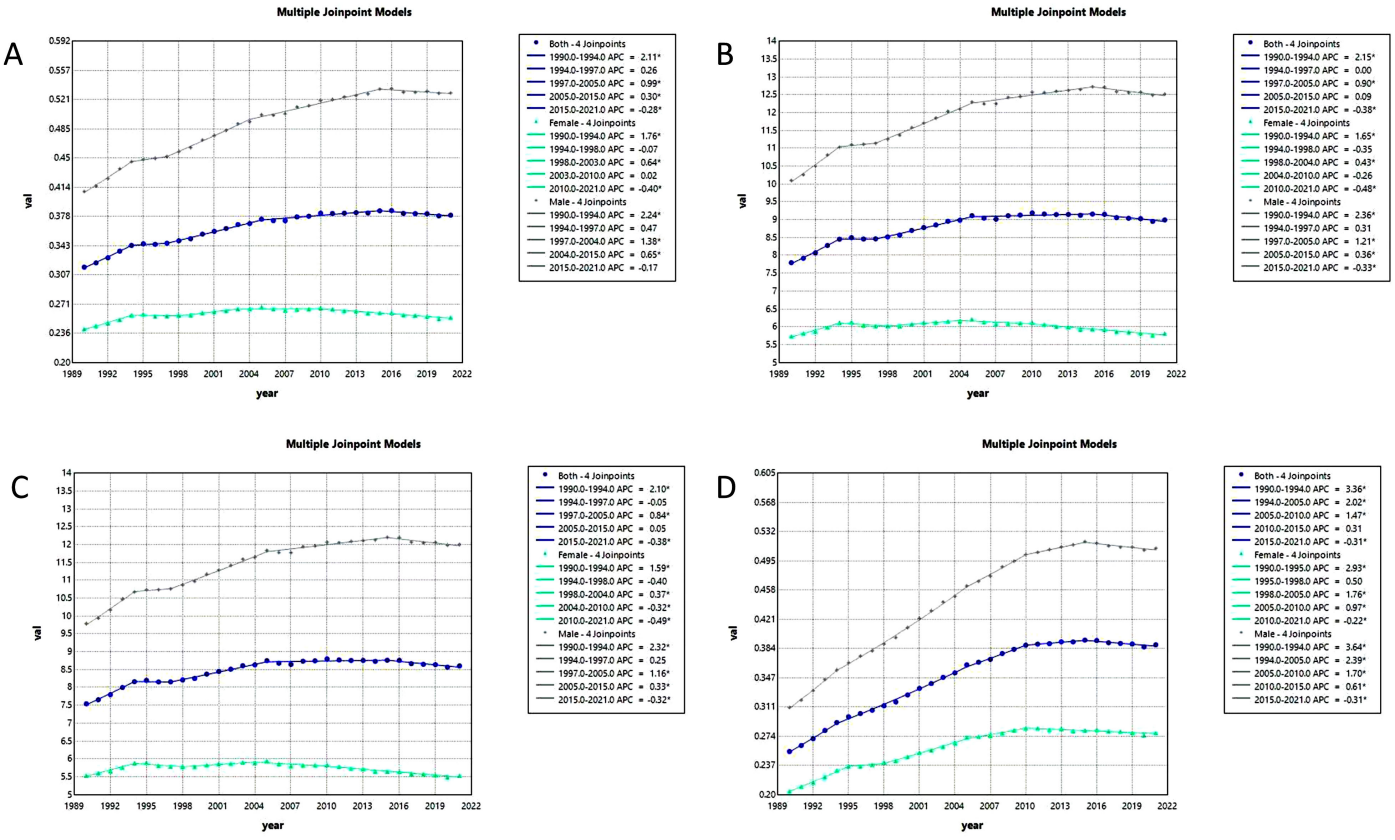
Joinpoint regression analysis of high BMI-associated kidney cancer burden temporal trends, 1990–2021. ASMR **(A)**, ASDR **(B)**, YLLs **(C)**, YLDs **(D)** for joinpoint regression analysis.

Joinpoint Regression analysis showed that ASMR and ASDR in kidney cancer from 1990 to 2021, but showed a decline in 2016. The YLLs and YLDs also showed a similar trend. The situation was roughly similar for male and female, and a greater burden of disease was evident for male. ASMR for kidney cancer generally increased from 1990 to 2021 (AAPC = 0.59%; 95%CI: 0.47% to 0.72%; P<0.001), but annual growth slowed, even negative between 2015 and 2021 (APC = -0.28%; 95%CI: -0.46% to -0.09%; P<0.01) (**Figure 6A** and **Supplementary Table 1**). ASDR showed a global upward trend (AAPC = 0.46%; 95%CI: 0.32% to 0.60%; P<0.001) with the fastest growth in 1990-1994 (APC = 2.15%; 95%CI: 1.76% to 2.55%; P<0.001) and negative growth in 2016-2021 (APC = -0.38%; 95%CI: -0.58% to -0.18%; P<0.001) (**Figure 6B** and **Supplementary Table 2**). Similarly, YLLs continued to rise from 1990 to 2021 (AAPC = 0.42%; 95%CI: 0.29% to 0.56%; P<0.001) and negative growth from 2016 to 2021 (APC = -0.38%; 95%CI: -0.57% to -0.18%; P<0.001) (**Figure 6C** and **Supplementary Table 3**). In comparison, YLDs grew rapidly in the early stage and slowed down in the later stage, showing negative growth in 2016-2021 (APC = -0.31%; 95%CI: -0.48% to -0.14%; P<0.01) and showed an overall upward trend from 1990 to 2021 (AAPC = 1.37%; 95%CI: 1.24% to 1.50%; P<0.001) (**Figure 6D** and **Supplementary Table 4**).

### 3.6 Age-Period-Cohort analysis

**Figure 7:**
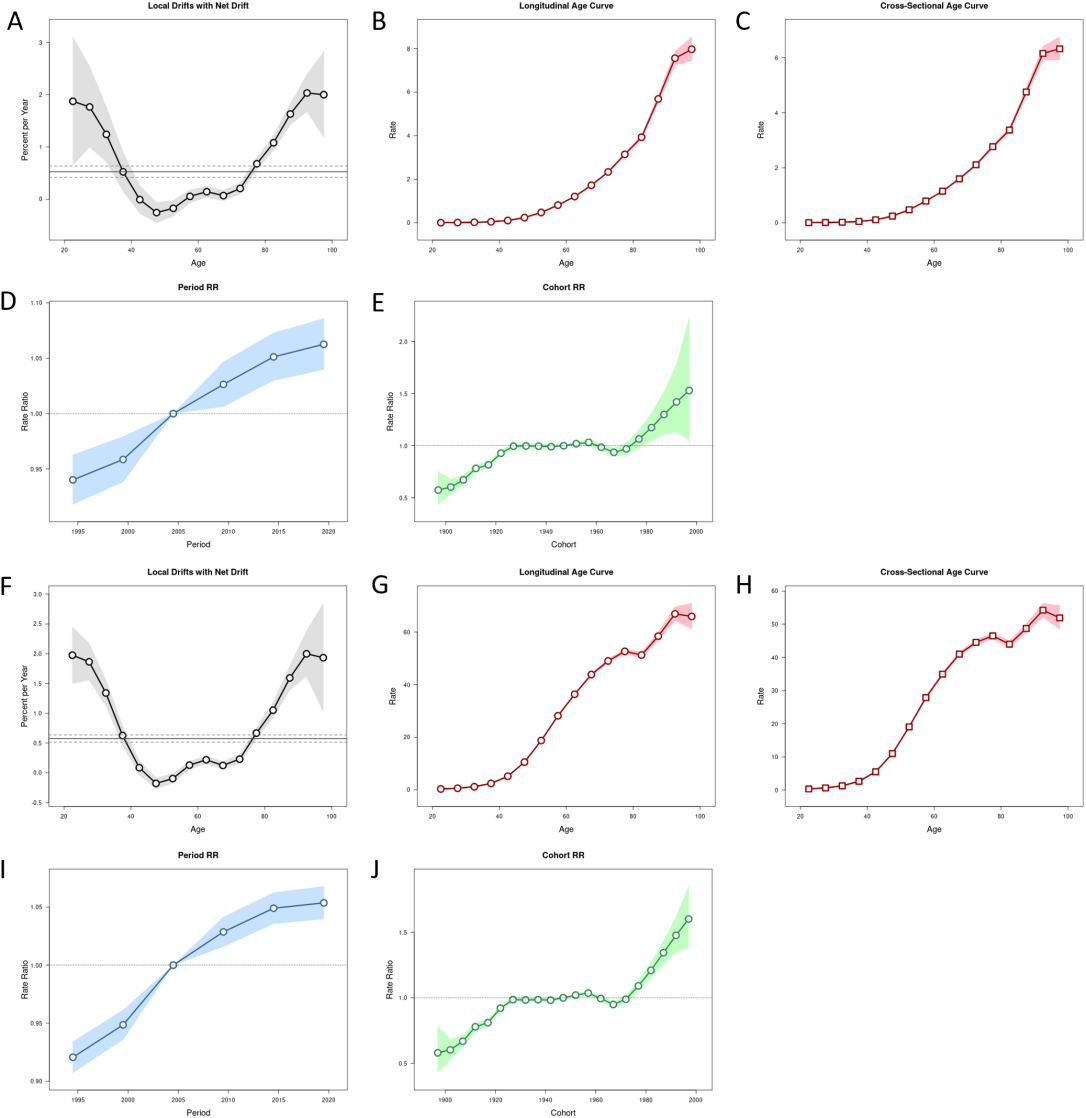
Age-Period-Cohort analysis results for deaths and DALYs. Local Drifts with Net Drifts **(A)**, Longitudinal Age Curve **(B)**, Cross-Sectional Age Curve **(C)**, Period RR **(D)**, Cohort RR **(E)** in deaths; Local Drifts with Net Drifts **(F)**, Longitudinal Age Curve **(G)**, Cross-Sectional Age Curve **(H)**, Period RR **(I)**, Cohort RR **(J)** in DALYs.

For Deaths, local drifts indicated significant annual increases in mortality among older age groups, aligning with the net drift toward higher burden in aging populations (**Figure 7A**). Age-specific curves highlighted a steep increase in mortality rates after age 40, peaking at ages 60–80, consistent with longitudinal and cross-sectional analyses (**Figures 7B, C**). The period effects of death showed a modest upward trend, with the RR increasing from less than 0.95 in 1995 to greater than 1.00 in 2020, suggesting that the effects of high BMI have been amplified in recent decades (**Figure 7D**). The cohort rate ratio (RR) had higher risk in the late birth cohort, with RR of 1.5 in the 2000s and lower risk in the early cohort (1990-1920) (0.5<RR<1.0) (**Figure 7E**).

For DALYs, Local drifts for DALYs were less pronounced but still signaled progressive annual increases (10–20% per year) across middle-aged populations (**Figure 7F**). Longitudinal age curves for DALYs displayed a bimodal distribution, with peaks at ages 40–60 and 80–100, underscoring the prolonged disability burden in older adults (**Figure 7G**). Cross-sectional curves further emphasized that rates plateaued at 60–80 years, indicating sustained health loss in this age bracket (**Figure 7H**). The period and cohort effect RR of DALYs also showed a rising trajectory similar to eaths (**Figures 7I, J**).

### 3.7 Frontier analysis and future prediction

**Figure 8:**
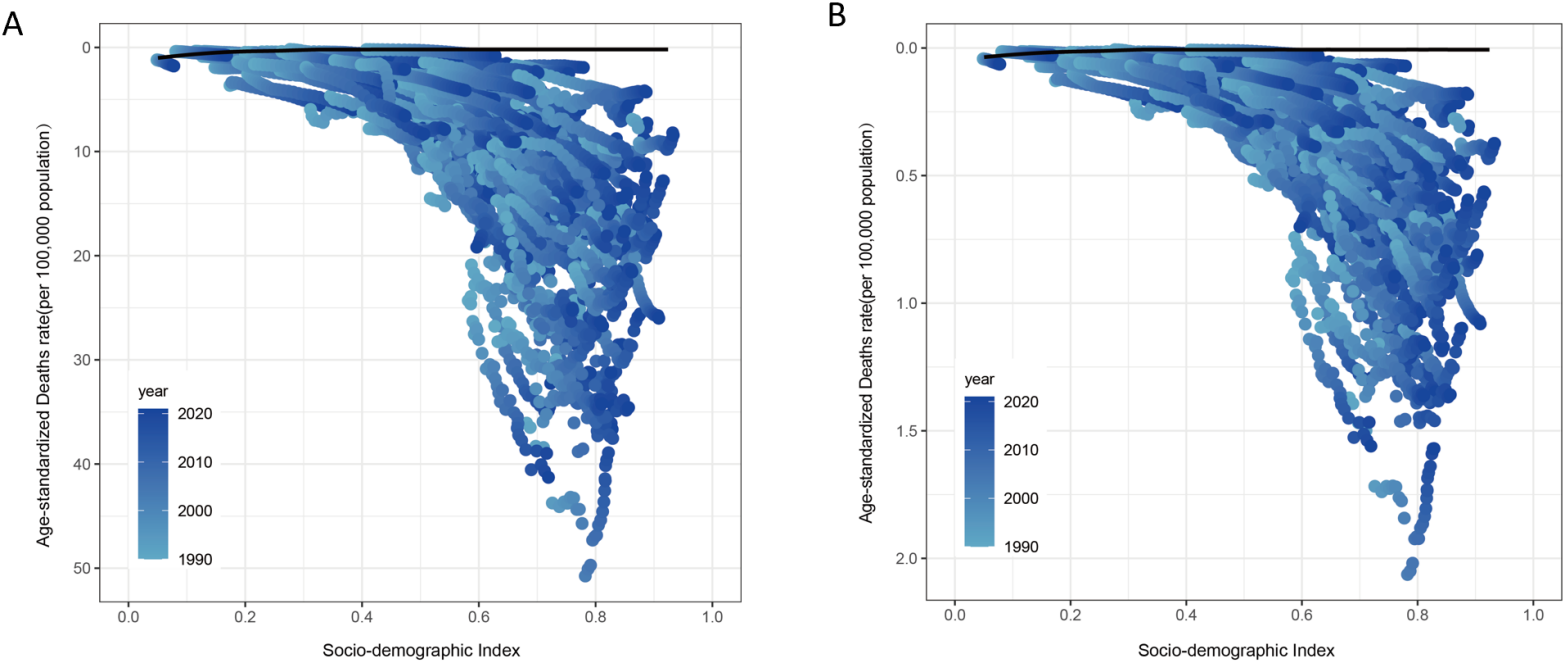
Trends in the association of the global burden of high BMI-associated kidney cancer with the SDI level, from 1990 to 2021. ASMR **(A)** and ASDR **(B)** for frontier analysis. The black line represented the frontier, and the dots represented the countries in this frontier analysis of the changing patterns.

Frontier analysis showed that ASMR and ASDR for high BMI-associated kidney cancer typically increase over time from 1990 to 2021, with the largest increase occurring between 2000 and 2010 (**Figures 8A, B**). There were significant differences between regions with different SDI levels. In regions with lower SDI levels, the growth rates of ASMR and ASDR showed a non-linear positive correlation with SDI and increased faster with SDI. Although areas with higher SDI levels had higher baselines, their growth rate slowed somewhat (**Figures 8A, B**).

**Figure 9:**
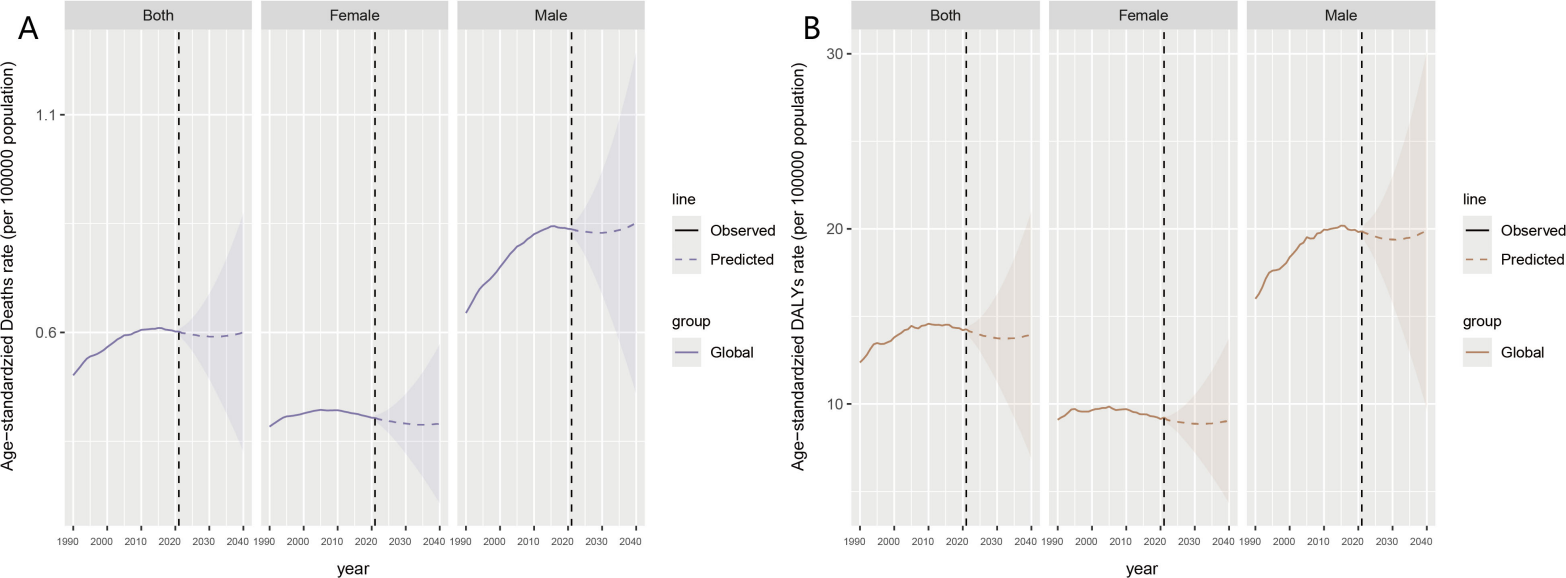
Future prediction by 2040 for high BMI-associated kidney cancer, both globally and by gender. ASMR **(A)** and ASDR **(B)** for future prediction. Observed data are shown as solid lines showing past data. The predicted data are represented as dashed lines showing future trend predictions. The shaded area represents the range of uncertainty in the forecast.

Future projections indicate that the global declining trends in ASMR and ASDR for high BMI-associated kidney cancer will continue until 2030, after which they are expected to rise again. For female, ASMR and ASDR have shown slight decreases and increases since 2021, overall remain relatively stable. In contrast, male rates have significantly decreased since 2021, with a more pronounced upward trend after 2030. male have consistently borne a higher disease burden, which is the primary reason for the increasing global burden (**Figures 9A, B**).

## 4 Discussion

The significant influence of obesity as a modifiable risk factor is highlighted by the rising incidence of kidney cancer worldwide. The findings of our study indicate that, in tandem with the rise in the prevalence of obesity worldwide (17), the number of deaths has climbed 2.67 times and the disability mortality rate has increased 66.1% between 1990 and 2021. While the ASR of kidney cancer will decline after 2016, the number of kidney cancer cases worldwide will continue to rise between 1990 and 2021. The burden of kidney cancer disease varies significantly by gender, with male bearing a greater burden than female and it is primarily found in areas with low SDI. These negative patterns could provide significant challenges to kidney cancer prevention and treatment.

The trends of various ages and genders demonstrate that the burden of kidney cancer rises with age, peaks in middle-aged and older populations, and varies significantly by gender. Over time, the middle-aged and older adult population has the highest mortality and disability rates from kidney cancer, with 60% of male deaths happening in those between the ages of 60 and 79. This is in line with middle-aged and older people’s cumulative exposure to metabolic diseases linked to obesity and age-related renal impairment. The burden of kidney cancer rises with age, according to the time trend, but the decrease in death and disability loss in the years following 2016 points to the possible advantages of better treatment approaches, such as immune checkpoint inhibitors and tyrosine kinase inhibitors that target the vascular endothelial growth factor receptor (VEGFR) (18), which have been shown to be effective in treating metastatic renal cell carcinoma. There is a clear gender imbalance in disease burden in most age groups. The gender disparity in obesity is a significant factor (19), albeit the possible causes are still unclear. More may also be explained by variations in genetic traits between genders. According to certain research (20, 21), gender has a separate role in kidney cancer survival and progression (22) (such as the protective effect of estrogen on renal cell carcinoma), and effects of social behavior (23) (such as low adherence to the obesity intervention and smoking in men). Therefore, when developing treatment plans for kidney cancer at the patient level and putting population-based preventative initiatives into practice, gender differences should be taken into account.

Regional variations were seen in the worldwide burden of kidney cancer linked to high BMI in 2021, and the annual growth rate of the disease was lower in high-burden regions. South America, North America, Europe, and North Asia are the regions with the highest disease burden, whereas sub-Saharan Africa, South Asia, East Asia, and Southeast Asia are the regions with the lowest disease burden. The treatment of obesity and the availability of healthcare resources and services may have an impact on disease burden. Increased imaging techniques have been linked to an increase in the incidence of kidney cancer in North American and European populations, which has been shown to contribute up to 50% to the inadvertent discovery of tiny renal tumors (24, 25). These areas do, however, also have a rather comprehensive health care system that may offer top-notch medical care, cutting-edge treatment plans, and aid in lowering death and raising survival rates. However, the efficient care and control of kidney cancer is limited in LMICs and areas due to their relatively limited resources in this field. The burden of kidney cancer rises with time due to a combination of factors such as population growth, particularly the aging of the population (26), and the rising incidence of chronic renal disease (27).

The link between the SDI and the disease burden of kidney cancer based on a high BMI is complex and non-linear. Because of their excessive calorie intake and sedentary lifestyle, people with moderate to high SDI are more likely to be obese (28–30). High-calorie foods are consumed more often since they are easy to prepare and affordable, which is in line with the rising obesity rates. On the other hand, because they make up a smaller percentage of the diet, people tend to eat fewer high-fiber items, such as fruits and whole grains, which raises their calorie intake and causes weight gain. This region lies between low and high levels of SDI and faces more unique challenges related to healthcare infrastructure and disease patterns, such as the transition from infectious to chronic diseases (31). The burden in high SDI areas is gradually being lessened thanks to significant advancements in rehabilitation programs and public health policy (32). The World Health Organization Committee on the Elimination of Childhood Obesity (33), which was established in 2014, made a number of recommendations in response to the growing trend of childhood obesity and overweight. WHO member states in Europe have responded by lowering obesity rates in line with this. The disease burden in regions with poor SDI is getting worse and increasing annually as a result of insufficient preventive efforts and delayed diagnostics in transitioning economies. Study’s (1) that used a Bayesian age cohort model predict that by 2030, kidney cancer incidence will increase in the majority of rising nations. These predictions for the upcoming ten years emphasize how urgently worldwide initiatives to combat obesity are needed, especially in areas that are quickly urbanizing. China has a huge population base, and even a smaller change in proportion can have a significant impact on health equality issues. From 1990 to 2021, the proportion of mortality and DALYs in China increased significantly, which emphasizes the dual challenges of addressing population growth and regional development.

Based on high BMI data, joint point regression analysis revealed that the burden of kidney cancer disease increased over time before declining after 2016. According to a 2019 study published by The Lancet Commission (35): obesity rates are rising globally; several evidence-based policy suggestions have not yet resulted in significant changes; Slow progress is caused by policy inertia, and obesity is frequently viewed as a singular problem. As a result, since 1990, the prevalence of kidney cancer disease has been steadily rising, with obesity serving as a risk factor. Age Sex Time Trends in Disease Burden and earlier related studies are in line with the declining trend in disease burden since 2016 (34), underscoring the possible advantages of better treatment approaches (18). Preclinical prevention and BMI reduction should receive more focus when kidney cancer clinical treatment is improved.

By exposing disparate impacts of aging, temporal patterns, and risk specific to a given birth cohort, the age-period-cohort analysis offers crucial insights into the temporal dynamics of high BMI-associated kidney cancer burden. The mortality and handicap loss from kidney cancer linked to high BMI increased dramatically after the age of 40 and peaked between the ages of 60 and 80. The increasing incidence of kidney cancer is consistent with population aging. The period impact emphasizes how the burden of kidney cancer increased gradually between 1990 and 2016 before somewhat declining after that year. This turning phase is in line with early identification initiatives in high regions and the worldwide rollout of targeted therapy (18). The fact that low-SdI areas had restricted access to these advancements, resulting in a persistent rising trend (34), emphasizes how healthcare disparities mediate risk for particular time periods. According to the cohort effect, the risk ratio (RR = 1.5) was considerably higher for those born in 2000 than for those born earlier (RR<1.0). This conclusion is consistent with shifting trends in eating habits and lifestyle choices brought about by economic globalization. The prevalence of ultra-processed diets and sedentary lifestyles, particularly in quickly urbanizing areas, is the cause of the elevated risk in younger generations. Together with decreased physical activity brought on by technology, these dietary changes increase obesity rates, which in turn increase the younger generation’s cumulative exposure to high Bmi-associated carcinogenic pathways. Crucially, there were noticeable variations in the interactions between the cohort and the SDI class. While the low-sdi cohort faced the complex risk of an unregulated food environment and delayed diagnosis, the high-sdi cohort born after 2000 demonstrated slower risk accumulation as a result of progressive public health policies [e.g., Mexican sugar tax (36), Scandinavian school nutrition programs (37)]. These results imply that different exposures to obesogenic environments caused by economic globalization worsen health disparities.

Frontier analysis reveals a rising trend in the burden of high BMI-associated kidney cancer, particularly from 2000-2010. Projections suggest future trends through 2040. Global kidney cancer burden fell from 2016-2030, then surged, suggesting short-term relief measures might be insufficient long-term. First, the projected resurgence may be attributed to demographic aging—particularly in regions with lower socioeconomic development—as well as the phenomenon often termed the “obesity paradox”. This paradox refers to observations that individuals with overweight or obesity sometimes exhibit better survival outcomes compared to those with normal BMI in certain disease contexts, including kidney cancer. For example, a large-scale study involving 50,717 patients (38) reported higher survival among heavier individuals, suggesting that elevated BMI might be linked to certain protective metabolic or nutritional reserves in the short term. Nevertheless, this apparent survival advantage may mask more complex underlying pathophysiological processes, and does not negate the established role of obesity in increasing cancer incidence. Over the longer term, the cumulative effects of population aging and sustained rises in obesity prevalence (39) may overwhelm any initial protective associations, ultimately reversing these patterns and contributing to growing disease burden. Second, healthcare access disparities may contribute significantly. High SDI regions maintain progress through advanced treatments, while low-to-middle SDI areas—with rising obesity and weaker medical systems—face delayed or inadequate interventions. This imbalance could postpone global recovery until post-2030, as early gains in affluent regions are countered by escalating burdens elsewhere. The initial decline likely stems from better early detection, surgical and systemic therapy advances (e.g., targeted and immunotherapy), and public health measures like diet and exercise promotion. To assess the potential bias from incidental diagnoses, a sensitivity analysis excluding regions with the highest rates of incidental detection (e.g., North America and Europe) could be conducted in future studies. Moreover, despite interventions, high BMI may still drive kidney cancer rates, as childhood overweight/obesity is projected to reach 30% globally by 2030 (boys: 34.2%, girls: 27.4%) (40), underscoring the continued challenge of obesity reduction, especially among males. Gender-specific data reveals men will face a disproportionately higher global burden, with rising male incidence rates post-2030 underscoring the need for targeted interventions. These findings emphasize the necessity of dual strategies to mitigate kidney cancer resurgence. To effectively address the rising burden of kidney cancer, a two-pronged approach is essential. First, we must strengthen preventive measures—implementing tighter food regulations, prioritizing obesity screening for men, and expanding community-based weight management programs. Targeted interventions could mitigate the projected rise in kidney cancer burden, particularly in middle- and low-SDI regions. Second, we need to guarantee fair and widespread access to diagnostic tools and treatments worldwide. Without these fundamental reforms, any short-term reduction in kidney cancer cases will likely prove fleeting rather than sustainable. Our projections are based on current epidemiological trends and do not account for potential disruptions such as global pandemics, major policy shifts, or breakthroughs in obesity or cancer therapeutics. Future studies should incorporate scenario-based modeling to better capture these uncertainties.

Our study systematically evaluated the global trend of high BMI-associated kidney cancer from 1990 to 2021, analyzed the spatial distribution characteristics, temporal trends and age-gender differences in 204 countries and regions. Finally, it makes a prediction for the future to 2040. Full-dimensional data coverage and multi-method integration enhance the conclusions’ resilience and offer accurate epidemiological support for regionally tailored preventive and control. There are various restrictions on this study. First, in areas where data is scarce, such as parts of sub-Saharan Africa and South Asia, cancer registrations are incomplete or non-existent, which poses big problems. In these places, data on kidney cancer caused by high BMI are not directly measured, but are derived from models-using information from other areas with sufficient data, so the results may be inaccurate. These models are based on assumptions such as the relationship between BMI and cancer risk and medical conditions that are the same globally, but these assumptions may not hold true locally. And because many cases are not diagnosed, the model is likely to underestimate the real burden. The same assumption affects global data: even a small amount of systematic error in low-SDI regions with large populations can distort global totals and trends. For example, if a “detection gap” arises-obesity rates have increased but diagnoses have not kept up-it may mislead people into thinking that the disease burden is increasing, or even lead to low future global estimates. Second, diagnostic misclassification (e.g., incidental testing and symptom testing) may influence temporal trends, especially in high-income areas. Third, although GBD was adjusted for high BMI attribution, residual confounding from unmeasured variables (e.g. genetic susceptibility or environmental exposure) cannot be excluded. And we adjusted for several confounders in the GBD model, factors such as smoking, hypertension, and occupational exposures (e.g., trichloroethylene) may interact with high BMI in influencing kidney cancer risk. Future studies should explore these interactions in more depth. Additionally, variations in health systems, diagnostic capabilities, and case definitions across countries may affect the accuracy and comparability of DALY estimates, particularly in cross-national comparisons. Finally, the Age-Period-Cohort model assumes a linear relationship of cohort effects, which may oversimplify the complex interplay between obesity and renal carcinogenesis. However, the GBD study provides us with global data and provides an unprecedented opportunity to investigate the global burden. Here are some suggestions based on our study. Individuals with high BMI should pay attention to consuming high fiber foods such as fruits and whole grains, combined with tobacco control measures to reduce the synergistic carcinogenic effect with BMI, particularly in men aged 55–79 years. Each country should impose restrictions on high-calorie advertising and promote community health campaigns within factories. Low and middle SDI areas should improve the diagnostic and treatment techniques, strengthen the early kidney cancer screening and awareness of chronic disease management, especially for the middle-aged and older adult population.

## 5 Conclusion

In summary, this study highlights the significant and growing burden of high BMI-associated kidney cancer, particularly among men aged 55–79 years. Although treatment advances led to a temporary decline after 2016, a rebound is projected after 2030 due to rising obesity rates, population aging, and persistent healthcare disparities. To alleviate this problem, we recommend gender-specific measures, such as targeted screening for men aged 55–79 in high-SDI areas, community weight loss programs in developing economies, and policies to ensure that everyone has equitable access to advanced diagnosis and treatment. Urgent global action is needed to address the dual challenges of obesity and kidney cancer control.

## Data Availability

The datasets presented in this study can be found in online repositories. The names of the repository/repositories and accession number(s) can be found in the article/**Supplementary Material**.
